# Investigation of the hyperperfusion phenomenon following carotid artery stenting using preoperative computed tomography perfusion imaging

**DOI:** 10.1007/s00701-025-06545-0

**Published:** 2025-05-26

**Authors:** Hiroyuki Yamamoto, Daisuke Maruyama, Masataka Nanto, Naoya Hashimoto

**Affiliations:** Department of Neurosurgery, Kyoto Prefectural University of Medicine Graduate School of Medical Science, Kyoto, Japan

**Keywords:** Hyperperfusion phenomenon, Hyperperfusion syndrome, Computed tomography perfusion imaging, Single photon emission computed topography, Carotid artery stenting

## Abstract

**Purpose:**

This study aimed to identify the most effective parameters of computed tomography perfusion imaging (CTP) using the Bayesian estimation to predict hyperperfusion phenomenon (HPP) risk after carotid artery stenting (CAS).

**Methods:**

We retrospectively analyzed 46 patients who underwent CAS with preoperative CTP and preoperative and postoperative ^123^I-labeled N-isopropyl-p-iodoamphetamine (^123^I-IMP) single photon emission computed tomography (SPECT) at rest, between April 2019 and March 2024. Patients were categorized into the HPP and non-HPP groups based on the postoperative asymmetry index (AI) of cerebral blood flow (CBF) on ^123^I-IMP SPECT. Relative ratios of CBF, cerebral blood volume (CBV), mean transit time (MTT), and time-to-peak (TTP) were calculated from preoperative CTP and compared between the two groups. Correlations among each CTP parameter, preoperative AI, and postoperative AI were assessed.

Receiver operating characteristic (ROC) analysis identified the most accurate CTP parameters for predicting HPP.

**Results:**

HPP occurred in four patients, with one developing cerebral hemorrhage. Significant differences were observed between the HPP and non-HPP groups in CBV (*p* = 0.001), MTT (*p* = 0.003), and TTP ratio (*p* = 0.011), and preoperative AI (*p* = 0.021). Among the CTP parameters and preoperative AI, the CBV ratio showed a positive correlation with the postoperative AI (*r* = 0.63, *p* < 0.01). The CBV ratio demonstrated the highest area under the curve (AUC) for predicting HPP (AUC = 0.95). However, after Benjamini–Hochberg correction, statistical significance was lost (adjusted *p* = 0.07).

**Conclusion:**

This study evaluated the predictive value of preoperative CTP using the Bayesian estimation method for identifying HPP risk after CAS. CBV ratio may serve as a potential parameter for predicting HPP.

**Supplementary Information:**

The online version contains supplementary material available at 10.1007/s00701-025-06545-0.

## Introduction

Cerebral hyperperfusion syndrome (CHS) is one of the most important complications associated with revascularization procedures and refers to neurological syndromes caused by elevated cerebral blood flow that greatly exceeds the demand on brain tissue. The hyperperfusion phenomenon (HPP) refers to elevated cerebral blood flow on imaging without symptoms and is known to be a significant risk factor for CHS [2, 3, 8, 9, 11, 15–18, 20, 24, 30, 32, 35]. Several factors, including advanced age, chronic hypertension, high-grade stenosis, and inadequate collateral circulation can predispose individuals to HPP and CHS [3, 7]. However, the predominant pathophysiological mechanism involves failure of normal cerebral autoregulation, resulting from impaired cerebrovascular reserve (CVR) [11, 22]. Preoperative assessment of the risk of HPP requires the evaluation of CVR via acetazolamide (ACZ)-challenged single photon emission computed topography (SPECT) using ^123^I-labeled N-isopropyl-p-iodoamphetamine (^123^I-IMP) [2, 3, 8, 18, 24, 30] but complications such as acute heart failure and pulmonary edema have been reported [8, 20, 23, 24]. Computed tomography perfusion imaging (CTP) allows for a straightforward assessment of brain perfusion through multiple parameters obtained using a contrast agent. The Bayesian estimation method is based on a probabilistic approach to generate a probability distribution for the CTP parameters [29]. This method is less sensitive to noise and further shows better accuracy and image quality; therefore, its application may enhance the detection of abnormal perfusion and infarcts [7, 10, 29]. This study aimed to identify the preoperative CTP parameters, derived using the Bayesian estimation method, which most effectively predict the risk of HPP after CAS.

## Materials and methods

### Patients

This study was approved by the Ethics Committee of Kyoto Prefectural University of Medicine Hospital (ERB-C- 2730). This study enrolled patients admitted to our institute between April 2019 and March 2024. Patients were considered symptomatic if they had experienced a stroke, amaurosis fugax, or a transient ischemic attack involving the ipsilateral carotid area within 180 days of the initial assessment [31]. Measurements of carotid stenosis (percentage determined based on diameter) were obtained based on digital subtraction angiography using the methodology outlined by the North American Symptomatic Carotid Endarterectomy Trial (NASCET) [23]. Symptomatic patients with > 50% stenosis were considered for revascularization. For asymptomatic patients, we applied a strict institutional criterion, consideringadvancements in medical treatment [1, 4], and included only cases with > 80% stenosis for revascularization. We included patients who underwent preoperative CTP and ^123^I-IMP SPECT at rest within 3 months before CAS and postoperative ^123^I-IMP SPECT at rest within a day of CAS. Patients with contralateral carotid artery stenosis or occlusion, and those who underwent staged CAS were excluded.

### Periprocedural management

All patients received dual antiplatelet therapy with the antiplatelet agents aspirin (100 mg) and clopidogrel (75 mg) for at least 1 week prior to the procedure. All procedures were performed under local anesthesia using the transfemoral approach, during which the activated coagulation time was maintained at > 250 s via an intravenous bolus injection of heparin. The procedures were performed as follows: an 8 Fr sheath was introduced into the femoral artery and a 4 Fr sheath was introduced into the femoral vein. After an 8 Fr balloon-guiding catheter was navigated into the common carotid artery on the affected side via the 8 Fr sheath and the balloon was inflated, the 8 Fr balloon-guiding catheter was connected to the 4 Fr sheath using an interposed blood filter. During the flow reversal, a distal filter protection device was advanced into the cervical segment of the ICA on the affected side. CAS was performed under the dual protection of flow reverse and distal filter. Postoperatively, blood pressure was managed using nicardipine, maintaining systolic blood pressure between 110 and 140 mmHg. Blood pressure monitoring was performed noninvasively rather than via arterial line placement. For the first 24 h after CAS, all patients were managed in the high-care unit with hourly blood pressure management. On postoperative day 1 and 2, patients were transferred to the general ward, where blood pressure was measured every 4 h. From postoperative day 3 onward, routine management was implemented, with blood pressure measured three times per day. If HPP was diagnosed via ^123^I-IMP SPECT on the day after CAS, blood pressure management was continued until a follow-up ^123^I-IMP SPECT at rest was performed on postoperative day 7 or 14. Blood pressure control was discontinued upon confirming the normalization of cerebral blood flow (CBF). Dual antiplatelet therapy was continued for at least 1 month, after which single antiplatelet therapy was administered.

### CTP protocol

All patients underwent preoperative CTP using a 320-detector row CT scanner (Aquilion ONE; Canon Medical Systems, Tokyo, Japan). CTP acquisition was performed using a dynamic contrast agent-enhanced scan covering the entire brain with a 0.5-mm section thickness, 512 × 512 matrix, and a 200-mm axial field of view. A bolus (35–50 ml) of an iodinated contrast agent (iopamidol, 370 mg/mL; FUJI Pharma Co., Tokyo, Japan) was injected through a power injector into the right antecubital vein at a rate of 4–5 ml/sec, followed by 25 mL saline. The scanning protocol was as follows: dynamic volume scanning was started 5 s after contrast injection at 80 kVP and 240 mA. Three seconds after the first scan, 16 intermittent scans at 1.5 s intervals were applied for the arterial to venous phase and four intermittent scans at 4 s intervals for the late venous phase with 80 kVP and 120 mA. The scanning speed was 1 s second per rotation, and the total scan time was 59 s. The volumetric CT dose index for the protocol was 192 mGy.

### SPECT Protocol

All ^123^IMP-SPECT at-rest studies were performed using a dual-head gamma camera (Discovery NM/CT 670, GE Healthcare, Waukesha, WI, USA) within 3 months as well as on the day after CAS. Regional CBF was measured using the graph plot method using ^123^I-IMP. ^123^I-IMP was rapidly infused at a dose of 185 MBq into the right cubital vein. Dynamic planar images of the chest and head were acquired using a 128 × 128 matrix at 2 s per frame for 60 frames, beginning a few seconds before the transvenous infusion of ^123^I-IMP. We subsequently obtained head SPECT images on a 64 × 64 matrix 18 min after the ^123^I-IMP injection, and continued for 24 min.

### CTP parameters

We collected CTP parameters using automated perfusion analysis software (Vitrea Workstation 7.11; Canon Medical Systems), and CTP data were transferred to a Vitrea workstation and analyzed using the Bayesian estimation method for postprocessing. The arterial input function (AIF) was manually set in the MCA M1 segment on the unaffected side and the venous output function was manually set in the superior sagittal sinus. Subsequently, maps of CTP parameters, including CBF, cerebral blood volume (CBV), mean transit time (MTT), and time-to-peak (TTP) were obtained.

### Data analysis using an automated ROI-determining software

**Fig. 1:**
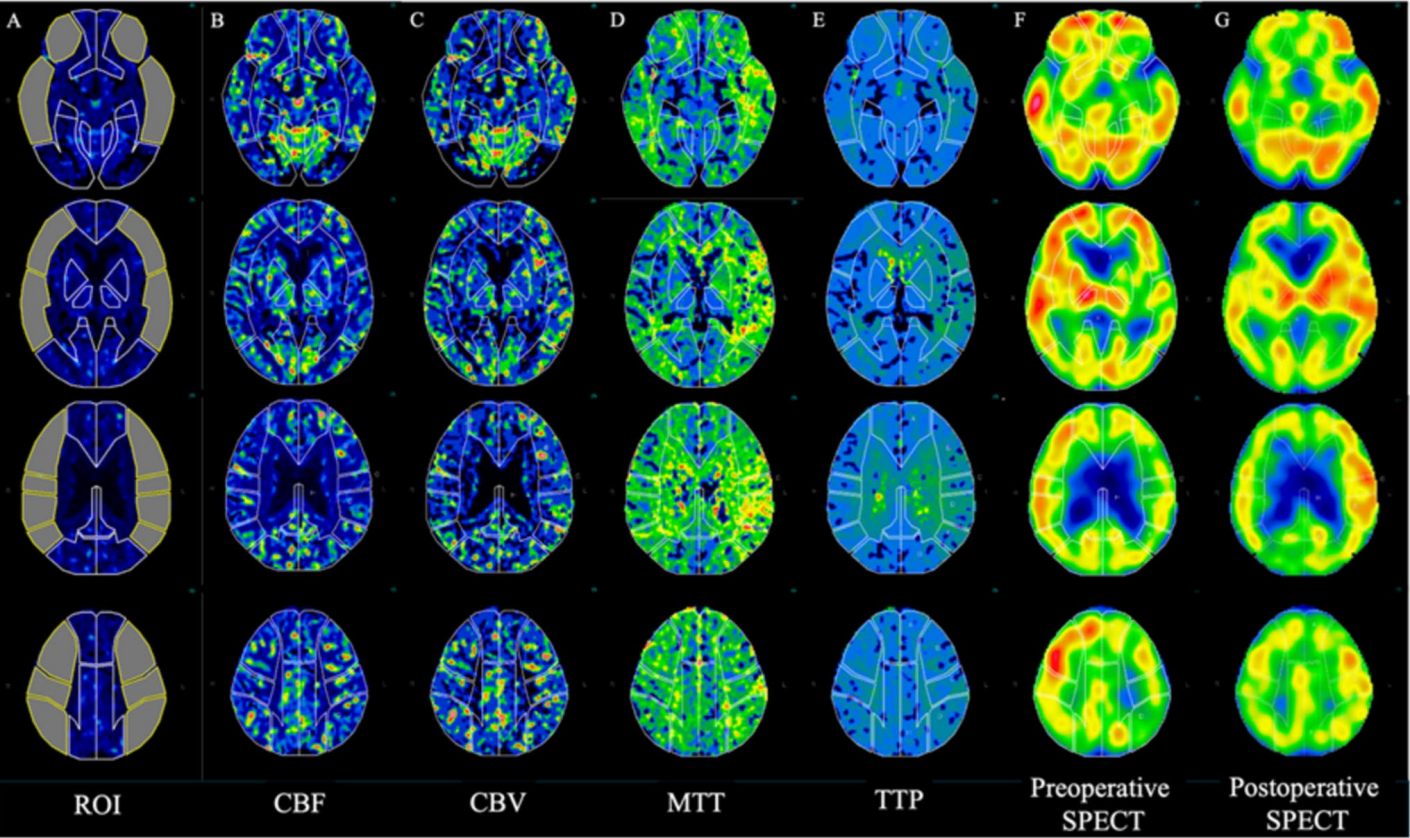
Reconstructed maps of CTP and ^123^I-IMP SPECT brain imaging at rest (case 10: left carotid artery stenosis). Regions of interest were automatically determined using 3D-SRT software, which converts volume data of CTP and ^123^I-IMP SPECT at rest to 59 slices of 2-mm interval standardized images. In this figure, 4 representative slices of 59 slices in each map of CTP parameter and ^123^I-IMP SPECT at rest are presented. **A**: yellow-circled ROIs were used for calculation as ROIs in the MCA territory, **B**: cerebral blood flow (CBF), **C**: cerebral blood volume (CBV), **D**: mean transit time (MTT), **E**: time to peak (TTP), **F**: preoperative ^123^I-IMP SPECT at rest, **G**: postoperative ^123^I-IMP SPECT at rest. The patient developed HPP without cerebral hyperperfusion syndrome including cerebral hemorrhage after CAS

We used automated region of interest (ROI)-determining software (three-dimensional stereotactic ROI template [3D-SRT]; PDRadiopharma, Tokyo, Japan) to calculate the regional values of CTP parameters and the CBF of ^123^I-IMP SPECT at rest, as previously described [7, 8]. We applied volume scanned data of CTP and ^123^I-IMP SPECT at rest to 59 standard slices of 2-mm slices. The 3D-SRT software uses predefined ROIs for the standard brain atlas, providing quantitative values of each regional CTP parameter and the regional CBF of ^123^I-IMP SPECT at rest for each of the following 12 regions on both the right and left sides: the callosomarginal, precentral, central, parietal, angular, temporal, posterior cerebral, pericallosal, basal ganglia, thalamus, hippocampus, and cerebellum. We determined the mean CBF, CBV, MTT, and TTP values of CTP, and the CBF value of ^123^I-IMP SPECT in the MCA territory using precentral, central, parietal, angular, and temporal ROIs, as previously described [7, 8]. We defined the ratios of the average CBF, CBV, MTT, and TTP values of CTP in the affected MCA territories to the unaffected side as the CBF, CBV, MTT, and TTP ratios respectively. We further calculated the asymmetry index (AI) as the ratio of the ipsilateral to contralateral CBF of ^123^I-IMP SPECT at rest in the MCA territories in the same way as each ratio of the CTP parameters. Figure 1 shows the reconstructed maps of CTP and ^123^I-IMP SPECT at rest in an example case. Prior studies have defined HPP in several ways, such as an increase of postoperative AI by > 4.6% or 6.1% [24, 25]. These studies established threshold for postoperative AI as the mean + 3 standard deviations (SDs) of the healthy control group. However, since we do not have data from a healthy control group, we defined HPP using more stringent criteria as postoperative AI≧1.1.

### Statistical analysis

Data were analyzed using R version 4.1.0 (The R Foundation for Statistical Computing). Continuous variables were presented as the mean and interquartile range. Categorical data were reported as frequencies (percentages). Comparative analyses of continuous and categorical variables between the HPP and non-HPP groups were performed using the Mann–Whitney U test and Fisher's exact tests, respectively. The CTP ratio and AIs were compared between the two groups using the Mann–Whitney U test. Correlations between each CTP ratio and the postoperative AI were assessed. Receiver operating characteristic (ROC) curve analysis was used to evaluate the predictive performance of the ratios of average CTP values obtained using the Bayesian estimation method for HPP. The area under the curve (AUC), sensitivity, specificity, positive predictive value (PPV), and negative predictive value (NPV) were calculated. Statistical significance of the AUC was evaluated using the bootstrap method, with the null hypothesis assuming AUC of 0.5. Optimal thresholds were determined based on the ROC operating point, assigning equal importance to sensitivity and specificity, with the overall accuracy reflected by the AUC. To account for multiple comparison, p-values were adjusted using Benjamini–Hochberg method, setting the false discovery rate (FDR) at 0.05 to prevent overestimation of statistical significance. Statistical significance was set at *p* < 0.05.

## Results

### Patient characteristics

**Fig. 2:**
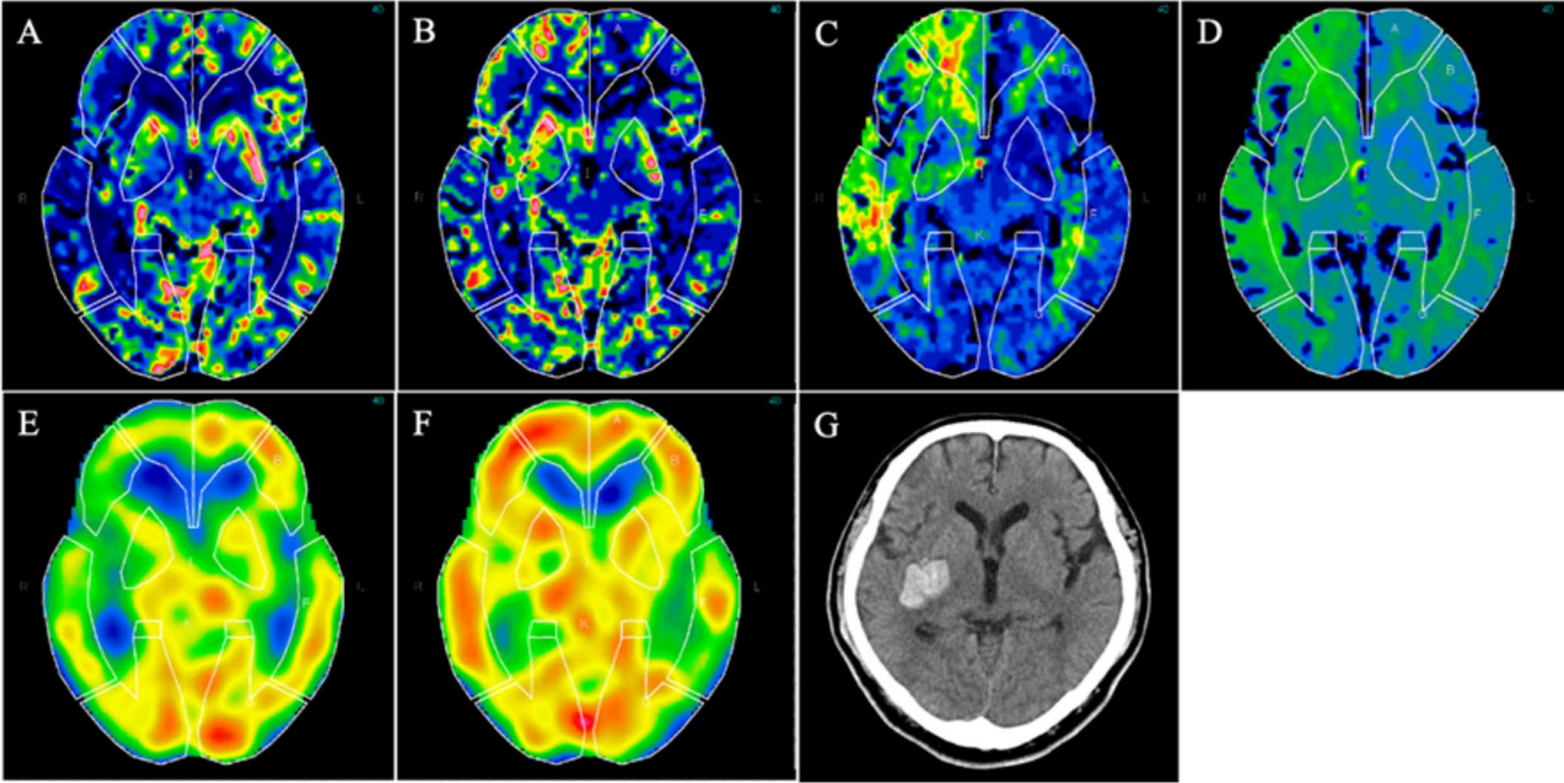
Imaging results of a 65-year-old man with a right carotid artery stenosis followed by intracerebral hemorrhage after CAS. Reconstructed maps of preoperative CTP showing a relatively decreased regional CBF (CBF ratio: 0.83) (**A**), increased regional CBV (CBV ratio: 1.24) (**B**), increased regional MTT (MTT ratio: 1.31) (**C**), and increased regional TTP (TTP ratio: 1.10) (**D**) in the right hemisphere. Preoperative ^123^I-IMP SPECT at rest showing decreased regional CBF (AI: 0.88) in the right hemisphere (**E**). Postoperative ^123^I-IMP SPECT at rest showing increased regional CBF (AI: 1.17) in the right hemisphere after CAS (**F**). The patient had cerebral hemorrhage in the ipsilateral basal ganglia on the postoperative day 3 after CAS (**G**)

During the study period, 220 consecutive patients with carotid artery stenosis were screened and 66 underwent CAS. Among them, four patients who did not undergo preoperative CTP, ^123^I-IMP SPECT at rest, or postoperative ^123^I-IMP SPECT at rest, nine with contralateral carotid stenosis or occlusion, and seven who underwent staged angioplasty were excluded. Finally, 46 patients with a mean age of 74.0 ± 8.1 years (range: 52–87 years) with unilateral carotid artery stenosis who underwent CAS with preoperative CTP and preoperative and postoperative ^123^I-IMP SPECT at rest were enrolled. The mean stenosis rate was 78.7 ± 12.5% (range: 65–95%). Twenty-three patients exhibited symptomatic ICA stenosis and 25 underwent CAS on the right side. Four of 46 patients (8.7%) developed HPP after CAS, of whom one (2.2%) further experienced cerebral hemorrhage in the ipsilateral basal ganglia on the postoperative day 3 (Fig. 2). Other patients with HPP did not experience any neurological symptoms during the postprocedural period. The clinical characteristics of the patients with and without HPP are presented in Table 1. Patients in the HPP group showed a tendency towards greater severity of carotid artery stenosis than those in the non-HPP group (*p* = 0.062).

**Table 1:** Clinical characteristics of patients in the groups with and without HPP

	No. of patients (%)		
Variable	without HPP (*n =* 42)	with HPP (*n =* 4)	*P* value
General characteristics			
Age (years)	76 (52–87)	71 (65–76)	0.439
Sex (male)	37 (88.1)	2 (50.0)	0.104
Risk factor			
Hypertension	37 (76.2)	3 (75.0)	1
Diabetes mellitus	30 (71.4)	4 (100)	0.56
Dyslipidemia	17 (40.5)	3 (75.0)	0.303
Smoking	28 (66.7)	3 (75.0)	1
Lesion characteristics			
Symptomatic stenosis	21 (50.0)	2 (50.0)	1
Stenosis rate (%)	77 (65–85)	91 (85–95)	0.062
Symptomatic side (rt)	22 (52.4)	3 (75.0)	0.614

*SPECT *single photon emission computed topography, *AI* asymmetry index, *CTP* computed tomography perfusion imaging, *CBF* cerebral blood flow, *CBV* cerebral blood volume, *MTT* mean transit time, *TTP* time to peak, *, *p* < 0.05

### Preoperative ^123^I-IMP SPECT and CTP parameters between HPP and non-HPP groups

Table 2 presents the AI of ^123^I-IMP SPECT and CTP ratio data between the HPP and non-HPP groups. The preoperative AI was significantly lower in the HPP group than in the non-HPP group (*p* = 0.021). The CBV, MTT, and TTP ratios were significantly higher in the HPP group than in the non-HPP group (*p* = 0.001, *p* = 0.003, and *p* = 0.011, respectively). The CBF ratio did not significantly differ between the groups (*p* = 0.227).

**Table 2:** Characteristics of SPECT and CTP parameters between the groups with and without HPP

	No. of patients (%)		
Variable	without HPP (*n =* 42)	with HPP (*n =* 4)	*P* value
SPECT			
preoperative AI	0.96 [0.87, 1.02]	0.91 [0.88, 0.94]	0.021*
postoperative AI	0.98 [0.90, 1.05]	1.11 [1.10, 1.17]	0.001*
CTP			
CBF ratio	0.95 [0.83, 1.06]	0.87 [0.83, 1.08]	0.227
CBV ratio	1.02 [0.85, 1.27]	1.25 [1.08, 1.49]	0.001*
MTT ratio	1.05 [0.92, 1.27]	1.31 [1.10, 1.51]	0.003*
TTP ratio	1.02 [0.98, 1.08]	1.10 [1.02, 1.14]	0.011*

*SPECT *single photon emission computed topography, *AI* asymmetry index, *CTP* computed tomography perfusion imaging, *CBF* cerebral blood flow, *CBV* cerebral blood volume, *MTT* mean transit time, *TTP* time to peak, *, *p* < 0.05

### Correlation between each CTP parameter and postoperative AI

**Fig. 3:**
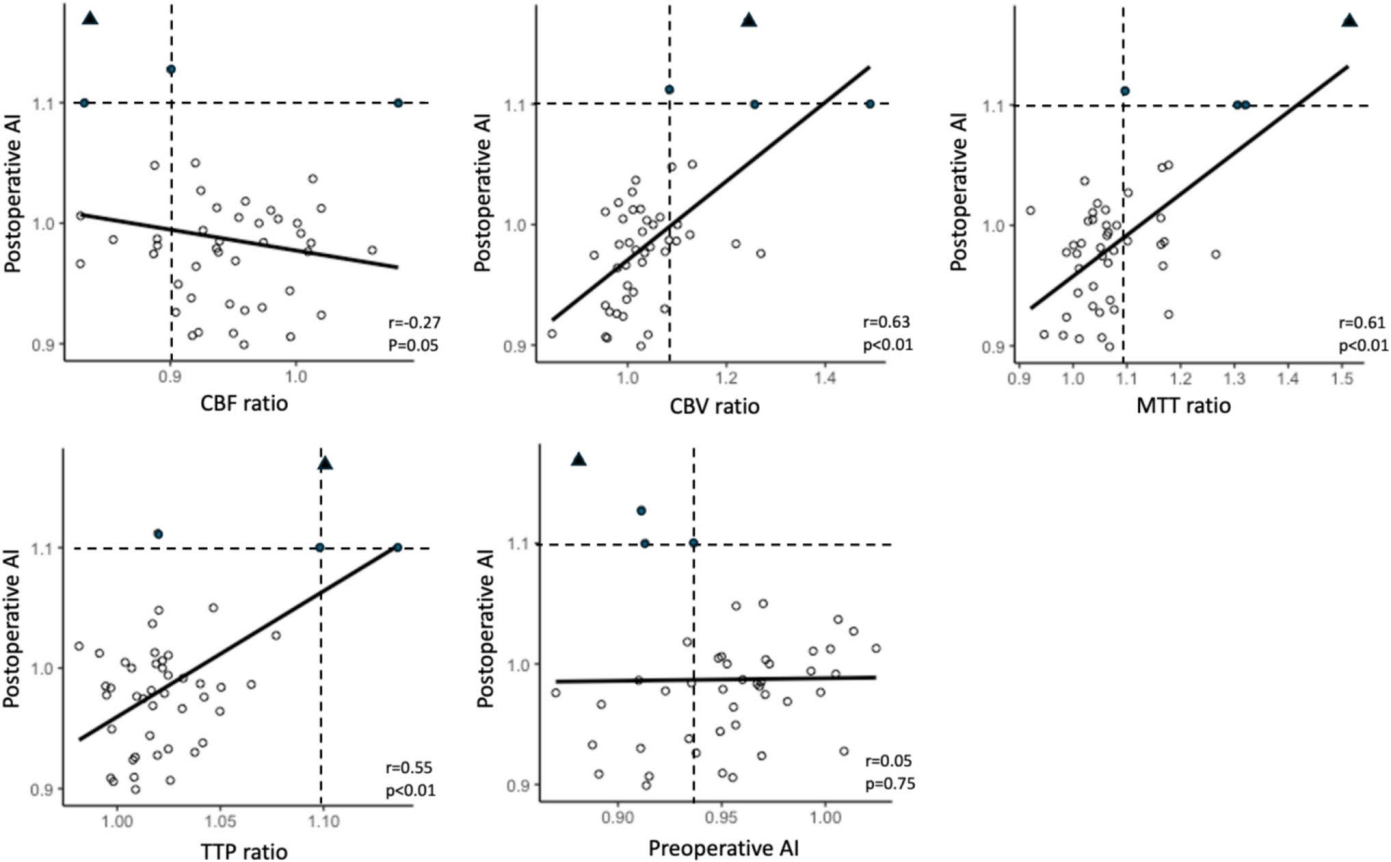
Scatter plots showing the correlation between each CTP ratio and postoperative AI. Black hollow circles (○) indicate the cases without HPP. Black filled circles (●) indicate cases with HPP, and black triangles (▲) indicate cases with HPP followed by intracerebral hemorrhage after CAS. The black line in each plot shows the regression line. Dashed lines show thresholds of each CTP parameter and postoperative AI to distinguish patients with HPP from patients without HPP

Figure 3 presents scatter diagrams and regression lines for each CTP parameter, preoperative AI, and postoperative AI. Moderate linear correlations were observed between the CBV ratio and postoperative AI (*r* = 0.63,* p* < 0.01), the MTT ratio and postoperative AI (*r* = 0.61, *p* < 0.01), and the TTP ratio and postoperative AI (*r* = 0.55,* p* < 0.01). A weak linear negative correlation was observed between the CBF ratio and postoperative AI (*r* = − 0.27, *p* = 0.05). No significant correlation was observed between the preoperative and postoperative AI (*r* = 0.05,* p* = 0.75).

### ROC analysis

**Fig. 4:**
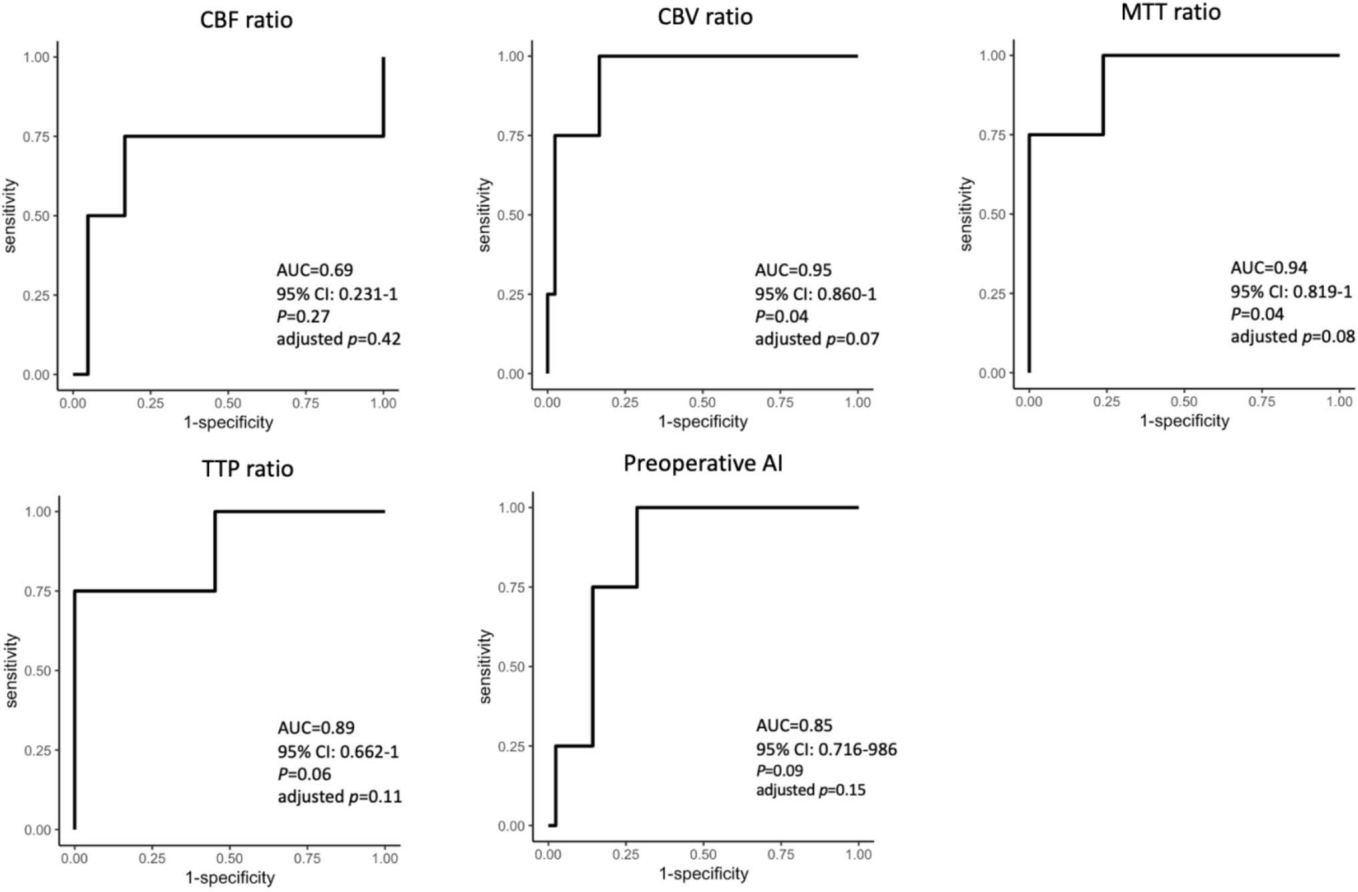
Receiver operating characteristic curves of the CBF ratio, CBV ratio, MTT ratio, TTP ratio, and preoperative AI for the identification of patients at risk for HPP

Figure 4 and Table 3 present the results of the ROC curve analysis and AUC calculation for each CTP parameter ratio and preoperative AI for the prediction of HPP. The CBV (AUC 0.95 [95% CI 0.86–1]), MTT (AUC 0.94 [95% CI 0.819–1],), and TTP (AUC 0.89 [95% CI 0.662–1]) ratios, as well as the preoperative AI (AUC 0.85 [95% CI 0.716–0.986]) all had AUC values > 0.8, whereases CBF ratio (AUC 0.69 [95% CI 0.231–1] did not. The CBV ratio was the parameter with the highest AUC for HPP, with which the optimal cutoff point of 1.085 (sensitivity 100%, specificity 83.3%). The PPV and NPV of the CBV ratio were 36.3% and 100%, respectively.

**Table 3:** Cutoff value, sensitivity, specificity, positive predictive value, and negative predictive value for HPP

Sensitivity, specificity, positive predictive value, and negative predictive value for HPP					
	Cutoff value	Sensitivity (%)	Specificity (%)	PPV (%)	NPV (%)
CBF ratio	0.900	75	83.3	30	97.2
CBV ratio	1.085	100	83.3	36.3	100
MTT ratio	1.096	100	76.2	28.5	100
TTP ratio	1.098	75	100	100	97.6
preoperative AI	0.937	100	71.4	25	100

*PPV* positive predictive value, *NPV* negative predictive value

The optimal cutoff points for the CBF, MTT, and TTP ratios, and preoperative AI were 0.900 (sensitivity 75%, specificity 83.3%), 1.096 (sensitivity 100%, specificity 76.2%), 1.098 (sensitivity 75%, specificity 100%), and 0.937 (sensitivity 100%, specificity 71.4%), respectively. The cutoff value, sensitivity, specificity, PPV and NPV of these factors are presented in Table 3.

Before multiple comparison correction,the AUCs of the CBV and MTT ratios (0.95 and 0.94, respectively) were statistically significant (*p* = 0.04, *p* = 0.04). However, after performed multiple comparison correction, the adjusted *p*-values were no longer statistically significant (adjusted *p* = 0.07, adjusted *p* = 0.08).

In the scatter plot showing the correlation between CBF and postoperative AI (supplement Fig. 1), the CBF ratio of 1.08 in the HPP group was considered as an outlier based on visual inspection. The case containing this point was excluded based on visual inspection because statistical outlier detection was deemed difificult due to the small sample size. Subsequently, ROC curve analysis and AUC significance testing were performed with multiple comparison correction (supplement Fig. 2). The AUCs of the CBV and MTT ratios decreased from 0.95 to 0.93 and from 0.94 to 0.92, respectively. No statistical significance was observed in both pre- or post-correction analyses (*p* = 0.05, *p* = 0.05, adjusted *p* = 0.09, adjusted *p* = 0.10). The cutoff values, sensitivity, specificity, PPV, and NPV of each parameter after outlier exclusion were presented in supplement Table 1.

### Clinical course of theree patients with HPP and a patient with CHS

In this study, four patients exhibited HPP on ^123^I-IMP SPECT at rest the day after CAS. The Postoperative AIs of three patients with HPP were 1.1, 1.1, 1.12, while the patients who developed HPP followed by intracerebral hemorrhage had a postoperative AI of 1.17. All patients were managed with intravenous nicardipine to maintain systolic blood pressure below 140 mmHg. Three of the four patients remained asymptomatic, experiencing no headache, seizures, or neurological symptoms such as hemiparesis after HPP diagnosis. ^123^I-IMP SPECT at rest performed on postoperative day 7 confirmed the normalization of CBF in the affected hemisphere, allowing for the discontinuation of blood pressure management. They were discharged between postoperative days 10 and 12 with a modified Rankin Scale score of 0. One of the four patients, who had symptomatic right-sided internal carotid artery stenosis (NASCET 85.5%) maintained good blood pressure control, but experienced sudden dysarthria and left hemiparesis on postoperative day 3. A head computed tomography scan revealed right basal ganglia hemorrhage (Fig. 2). ^123^I-IMP SPECT at rest performed on postoperative day 7 still showed increased CBF in the affected hemisphere; therefore, blood pressure management was continued. ^123^I-IMP SPECT at rest performed on postoperative day 14 confirmed the normalization of CBF, and blood pressure management was discontinued. The patient had persistent dysarthria and left hemiparesis, and was discharged on postoperative day 28 with a modified Rankin Scale score of 4.

## Discussion

The reported incidence of CHS ranges from 0.2% to 18.9% [2, 3, 18, 24, 32, 35], although variations in its definition (sometimes including HPP, which lacks standardization) make identifying the exact incidence difficult. Recently, the incidence of procedure-related complications, including cerebral embolism and postoperative ischemic stroke in CAS, has decreased to equivalent to that in carotid endarterectomy (CEA) due to the maturity of the procedure and development of devices in patients with symptomatic and asymptomatic carotid artery stenosis [19, 34]. A recent large registry reported that the incidence of CHS also decreased to 1.4%, suggested to be related to preventive strategies, such as staged angioplasty. However, when it does occur, 5.8% of patients die and 40.6% have major morbidity [33]. Despite the relatively low incidence of CHS after CAS, HPP can progress to CHS, including intracranial hemorrhage, which leads to considerable morbidity and mortality. [2, 3, 8, 18, 24, 32, 35] Moreover, several studies have indicated that postoperative hyperperfusion causes prolonged cognitive decline in patients who undergoing CEA [24, 25] Therefore, assessing the risk of HPP prior to CAS is crucial. This study evaluated the predictive utility of preoperative CTP using the Bayesian estimation method in conjunction with ^123^I-IMP SPECT at rest, to identify patients at risk of developing HPP following CAS. The findings demonstrated that the CBV, MTT, and TTP ratios were elevated significantly in patients with HPP compared with those without HPP. These parameters correlated postitively or negatively with postoperative AI, with the CBV ratio exhibiting the highest AUC for HPP prediction. Although ROC analysis did not reach statistical significance after multiple comparison correction, to the best of our knowledge, this is the first report to examine the utility of preoperative CTP using the Bayesian method for predicting HPP after CAS.

Reduced CBF alone does not necessarily indicate impaired CVR in the affected cerebral hemisphere [15, 17], because reduced CBF encompasses two distinct pathophysiological conditions: impaired CVR and matched hypometabolism resulting from cerebral ischemic lesions. In matched metabolism, CBF reduction is accompanied by decreased cerebral metabolism, which does not indicate an impaired CVR or an elevated risk of hyperperfusion following CAS. In the present study, the preoperative AI was below the cutoff value in all HPP cases, which could be interpreted as a feasible indicator for HPP prediction. However, the CBV ratio had the highest AUC value and a superior PPV to the preoperative AI. Furthermore, CBV showed a more significant correlation with the postoperative AI than with the preoperative AI. When the criteria for HPP were redefined as postoperative AI > 1.05, the CBV ratio could predict all cases of HPP; however, that for preoperative AI could not, as shown in Fig. 3. Although the CBV ratio did not archeive statistical significance after multiple comparison correction, its high AUC of 0.95 suggests potential clinical utility. Even after outlier exclusion, the AUC remained high (0.93), indicating minimal variability. The small sample size and low HPP incidence in this study likely contributed to the low PPV. However, considering its sensitivity, specificity, and AUC, these results also support the notion that the CBV ratio is a potentially useful predictor of HPP after CAS. Notably, in one case of HPP complicated by intracerebral hemorrhage, the CBV ratio did not show the highest value; while, preoperative AI showed the lowest value. This suggests that the CBV ratio alone was insufficient to fully explain the HPP severity or the risk of intracerebral hemorrhage. Further studies are needed to validate these hypotheses.

Previous PET studies have demonstrated that patients with occlusive carotid disease exhibiting an elevated oxygen extraction fraction and increased CBV are at a higher risk of subsequent ischemic stroke [6]. However, most CTP studies have suggested that MTT or TTP is more effective than CBV for evaluating impaired CVR or identifying patients at risk of HPP or CHS [3, 5, 32, 35]. The majority of CBV is venous and the extent to which autoregulatory vasodilation contributes to an increased CBV may vary considerably. In addition, previous studies have indicated that absolute CTP measurements commonly fail to yield precise results because of the inherent limitations of computational models compared with PET [3, 12]. Consequently, CBV has traditionally been regarded as less suitable for assesing impaired CVR or predicting HPP or CHS in CTP studies. However, our study revealed that the CBV ratio was moderately correlated with the postoperative AI and had a higher AUC value than the MTT ratio in identifying patients at risk for HPP, with the two metrics being statistically not significant. This finding may be attributed to two key factors: First, the CBV values calculated using the Bayesian estimation method are more accurate than those derived from classical approaches such as singular value deconvolution (SVD). SVD methods are inherently susceptible to oscillations, noise, and tracer delays, as minor variations in time-density curve magnitude can lead to significant deviations in the residue function post-deconvolution. This results in underestimation of CBF and the overestimation of MTT, particularly in cases with a low signal-to -noise ratio [10, 11, 14, 26]. CBV estimation is generally considered more stable with the SVD method due to its integral nature, even when standard or circular SVD algorithms were used [10, 13], patricularly under the relatively high radiation doses as applied in this study. However, it remains influenced by the time-density curve shape. If oscillations, noise, or tracer delay distort the curve shape, CBV estimation may become unstable, particularly due to excessive regularization or variations in the integral value caused by AIF fluctuations [13]. Indeed, Kudo et al. previously reported that delay dependency in CTP deconvolution methods could affect CBV, CBF, and MTT parameters [9]. In contrast, the Bayesian estimation method, which employs a probabilistic modeling framework and posterior distribution estimation, is less affected by these factors, leading to a more stable CBV estimation than the SVD method [13]. Therefore, the Bayesian estimation method, by mitigating the inherent limitaions of SVD, likely provides a more accurate CBV measurement, better reflecting CBV elevation associated with impaired CVR. Additionally, our analysis utilized volumetric data from 320-detector row CT, processed using automated ROI-determining software, to enable a more objective evaluation of perfusion parameters. Prior studies have further noted that the limited spatial coverage of traditional CTP techniques may fail to detect the areas most severely affected by stenoses [5, 9]. In contrast, whole-brain data from 320-detector row CT enabled a comprehensive analysis. Although quantitative data can be collected from brain CTP, previous studies have shown that the accuracy of absolute values is problematic due to limitations of the underlying models. Furthermore, AIF selection and ROI placement can vary between investigators, thereby affecting the consistency of the findings [3, 27]. To address these challenges, we manually set the AIF in the contralateral M1 and used the calculated ipsilateral-to-contralateral parameter ratios for analysis including the CBF, CBV, MTT, and TTP ratios, derived using automated ROI-based software. Since these methods are valid under the assumption that the cerebral perfusion of the contralateral hemisphere is normal, we excluded patients with contralateral carotid artery stenosis or occlusion. This approach ensured a valid and consistent comparison of the same ROIs between CTP and ^123^I-IMP SPECT at rest, thereby reducing bias and enhancing objectivity [7, 30] We propose that our method more accurately captures CBV elevation in the affected hemisphere due to impaired CVR compared than previous studies. Furthermore, it provides a more reproducible and clinically feasible approach for semi-quantitative preoperative CTP assessment brfore CAS.

The ACZ-challenged test remains a useful preoperative examination for detecting poor CVR and is widely used before surgery for ischemic cerebrovascular disease such as moyamoya disease and carotid artery stenosis [8, 21, 24, 25, 28]. Generally, in the preoperative evaluation of patients with carotid artery stenosis, the ACZ-challenged test is recommended only for cases suspected of having reduced CVR [8, 21, 24, 25] whereas it is routinely performed in moyamoya disease due to its relatively lower risk of atherosclerosis in younger patients. In contrast, carotid artery stenosis is more common in older patients with multiple comorbidities and a higher atherosclerosis risk. The systemic vasodilatory effects of ACZ can increase the risk of fatal complications such as heart failure and pulmonary edema [8, 21, 24, 25]. Therefore, the test is selectively conducted in high-risk cases [8, 19, 20]. In this study, the ACZ-challenged test was not performed, so a direct comparison with CT perfusion (CTP) parameters could not be made. Based on our results, we do not intend to alter our clinical routine for preoperative testing in carotid artery stenosis. However, considering that HPP did not occur in cases under with the CBV and MTT ratios below the cutoff values obtained via CTP using the Bayesian estimation, it is possible that the ACZ-challenged test could be omitted in such cases. We believe that CTP using the Bayesian estimation method has clinical implications for preoperative testing in CAS and plan to conduct future studies comparing the ACZ-challenged test with CTP using the Bayesian estimation method.

This study had several limitations. First, it involved a small cohort of patients, and was conducted as a single-center retrospective study. Futher investigations with larger, multicenter cohorts are necessary to confirm and generalize the study findings. Second, we observed significant time intervals between preoperative CTP and ^123^I-IMP SPECT at rest in some patients, during which time their clinical conditions may have changed. Consequently, we could not determine whether brain perfusion could be assessed under identical conditions using these two modalities. Third, we defined HPP using a criteria as postoperative AI≧1.1 without a statistical definition. However, after establishing 1.1 as the HPP positivity threshold, the mean and SD for the non-HPP group were calculated as 0.974 and 0.04 respectively, resulting in a mean + 3 SDs value of 1.094. We considered the 1.1 threshold setting to be largely acceptable, while the result was obtained post hoc. Fourth, limitation in data extraction from the default program of the Vitrea workstation prevented evaluation of Tmax, which is widely regarded as one of the most reliable and theoretically accurate metrics. Finally, this study did not incorporate ACZ-challenged ^123^I-IMP SPECT to assess CVR. Therefore, to validate our hypothesis of usefulness of CTP using the Bayesian estimation method for predicting HPP, future research should focus on comparing the CVR with CTP parameters derived via the Bayesian estimation method to improve HPP risk stratification.

## Conclusions

Overall, this study investigated the usefulness of preoperative CTP using the Bayesian estimation method for predicting the risk of HPP after CAS. The CBV ratio may serve as a potential candidate parameter for predicting HPP after CAS. However, because this was a preliminary study, further data collection in a prospective study is required to confirm the clinical relevance of these values in predicting HPP after CAS.

## Supplementary Information

Below is the link to the electronic supplementary material.

Supplementary file1 (DOCX 447 KB)

## Data Availability

The datasets analyzed during the current study are available from the corresponding author on reasonable request.

## Abbreviations

ACZ: Acetazolamide
AI: Asymmetry index
AIF: Arterial input function
AUC: Area under the curve
CAS: Carotid artery stenting
CBF: Cerebral blood flow
CBV: Cerebral blood volume
CEA: Carotid endarterectomy
CHS: Cerebral hyperperfusion syndrome
CTP: Computed tomography perfusion imaging
CVR: Cerebrovascular reserve
HPP: Hyperperfusion phenomenon
MTT: Mean transit time
NPV: Negative predictive value
PPV: Positive predictive value
ROC: Receiver operating characteristic
ROI: Region of interest
SD: Standard deviation
SPECT: Single photon emission computed tomography
SVD: Singular value decomposition
TTP: Time to peak
^123^I-IMP: ^123^I-labeled N-isopropyl-p-iodoamphetamine
