# Supplementary material for: Investigation of the hyperperfusion phenomenon following carotid artery stenting using preoperative computed tomography perfusion imaging
Source: Acta Neurochir (Wien). 2025 May 26;167(1):155. doi: 10.1007/s00701-025-06545-0 (PMC12106584; doi:10.1007/s00701-025-06545-0)
Supplement: Supplementary file 1 — Supplementary file1 (DOCX 447 KB) [file 701_2025_6545_MOESM1_ESM.docx]

**Supplement Fig 1** The outlier case (case 24) for each parameter is marked with a red arrow pointing a black filled circle. Within the scatterplot illustrating the relationship between CBF ratio and postoperative AI, a data point of a CBF ratio of 1.08 within the HPP group was identified a potential outlier. We identified the case as an outlier. The graphical elements, such as plot shapes and axis scales are identical to those in Figure 1 of the main text.


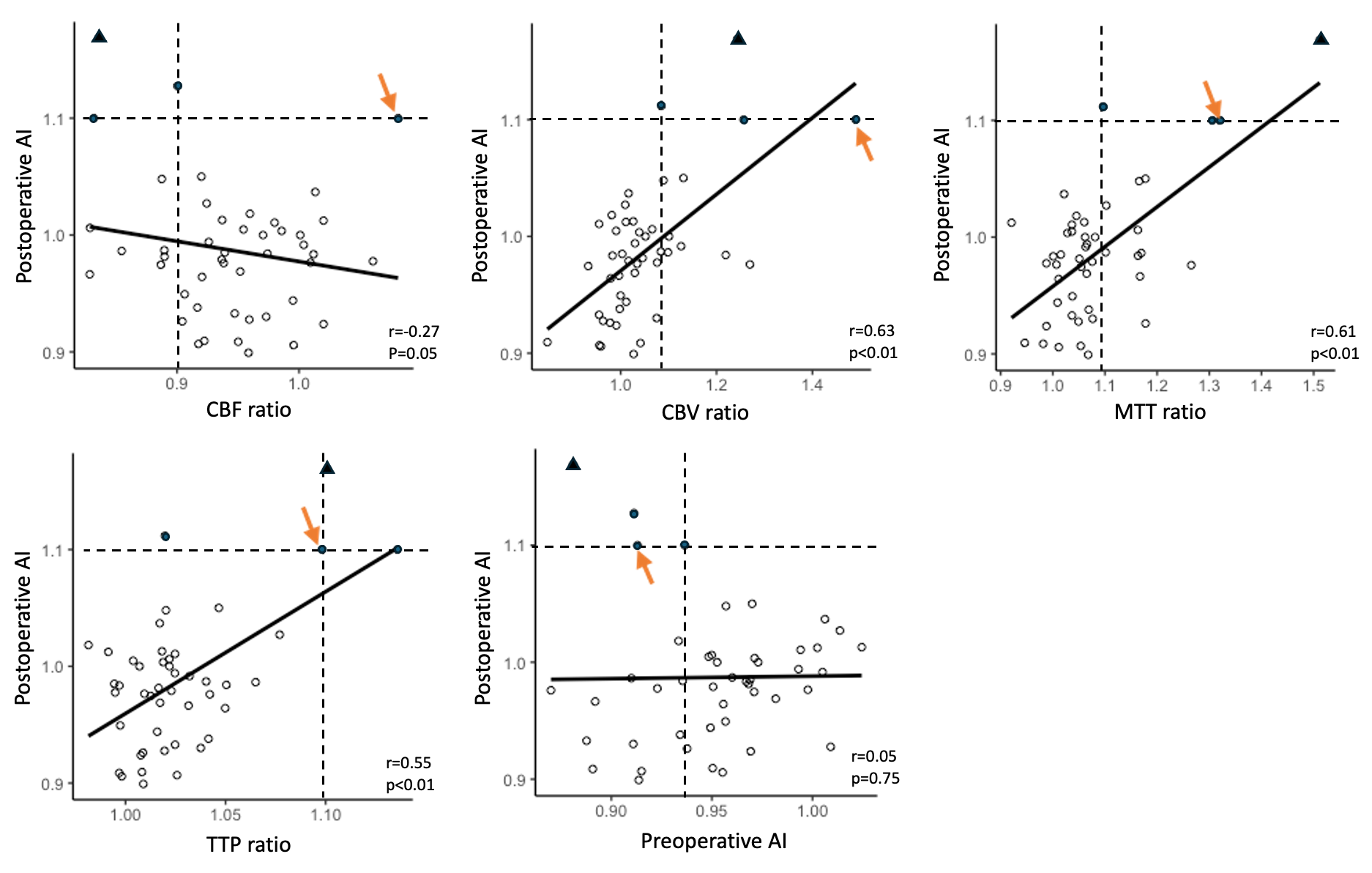


**Supplement Fig 2** Receiver operating characteristic (ROC) curves with AUCs and 95% confidence intervals of each CTP ratio and preoperative AI after outlier exclusion


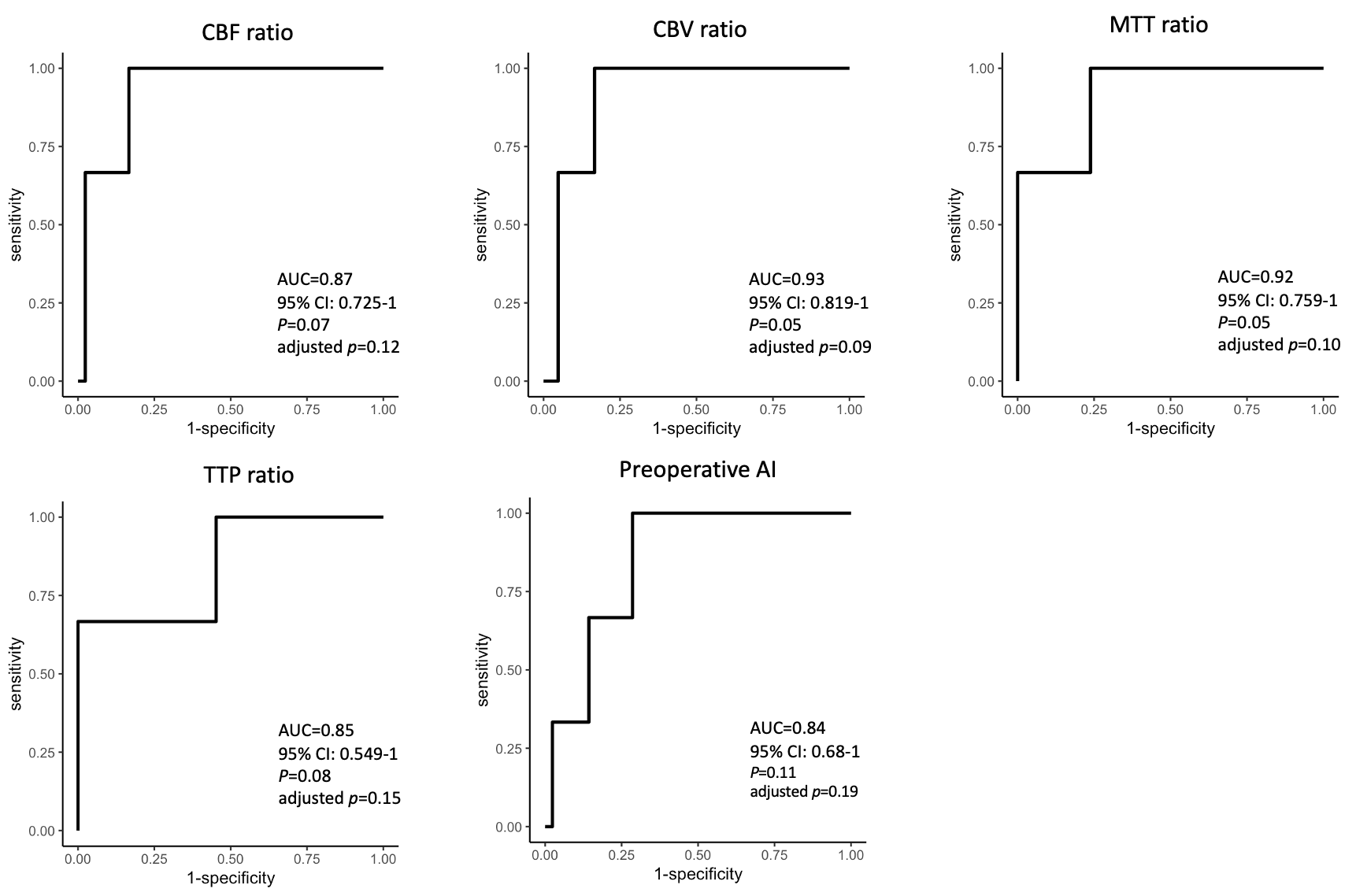


**Supplement Table 1** Cutoff value, sensitivity, specificity, positive predictive value (PPV), and negative predictive value (NPV) for HPP after outlier exclusion

| Sensitivity, specificity, positive predictive value, and negative predictive value for HPP | | | | | |
| --- | --- | --- | --- | --- | --- |
|  | Cutoff value | Sensitivity (%) | Specificity (%) | PPV (%) | NPV (%) |
| CBF ratio | 0.900 | 100 | 83.3 | 30 | 100 |
| CBV ratio | 1.084 | 100 | 83.3 | 30 | 100 |
| MTT ratio | 1.088 | 100 | 76.2 | 23 | 100 |
| TTP ratio | 1.089 | 67 | 100 | 100 | 97.6 |
| preoperative AI | 0.937 | 100 | 71.4 | 20 | 100 |
| PPV: positive predictive value, NPV: negative predictive value | | | | | |
